# Impact of Travel Distance and Transportation Needs on Racial/Ethnic Differences in Achieving Sustained Viral Suppression in Miami-Dade County, 2017

**DOI:** 10.21203/rs.3.rs-7049397/v1

**Published:** 2025-07-14

**Authors:** Rahel Dawit, Mary Jo Trepka, Dustin T. Duncan, Semiu O. Gbadamosi, Tan Li, Stephen Pires, Robert A. Ladner, Diana M. Sheehan

**Affiliations:** Florida International University; Florida International University; Columbia University; Florida International University; Florida International University; Florida International University; Behaivoral Science Research; Florida International University

## Abstract

Travel distance to receive HIV services and lack of reliable means of transportation may present a barrier to service utilization and influence HIV care outcomes. This study examined the moderating effect of travel distance and transportation needs to HIV medical case management (MCM) sites and AIDS Drug Assistance Program (ADAP) pharmacies on the association between race/ethnicity and sustained viral suppression. Data were obtained from the Miami-Dade County Ryan White Program, the American Community Survey, and Simply Analytics. Distance from the residences of the Ryan White clients to MCM sites and ADAP pharmacies were first calculated using the Network Analyst Tool within ArcGIS. We conducted multilevel logistic regression models to assess the role of transportation and travel distance on the association between race/ethnicity and sustained viral suppression. Most clients (88.9%) did not use the nearest MCM facilities to their residence. Among clients who did not have access to transportation (both public and personal), the odds of achieving sustained viral load suppression were lower for non-Hispanic Blacks (NHB) (adjusted odds ratio:0.28; 95% confidence interval:0.10–0.76), Hispanics (0.35; 0.13–0.94), and Haitians (0.16; 0.05–0.52) compared to non-Hispanic Whites. Also, Haitians and NHB had poor sustained viral suppression across other travel-related variables, including travel to the nearest facility of care, median travel distances to clients’ MCM site of choice, and median travel distance to the nearest ADAP pharmacy. Findings indicate that lack of access to transportation exacerbates racial/ethnic disparities in sustained viral suppression. Hence, it is important to address transportation needs and understand why clients obtain care from MCM sites that are not closest to their residence.

## Introduction

Access to transportation and geographic proximity to care facilities and pharmacies are important structural factors that may need to be addressed to maintain high levels of HIV medication adherence and suppressed viral load levels. Long travel distances and long travel times to receive HIV case management and pharmacy services may contribute to income-based, geographic, and racial/ethnic disparities in HIV care outcomes.^1, 2^ While most studies focused on the impact of travel distance in rural settings,^3, 4^ other studies have noted the influence of distance in urban settings.^5, 6^ The association is inconsistent,^7^ however, indicating that factors other than distance alone may influence a person’s choice of service site.^8^ These factors may include perceived stigma, provider-client relationship, reputation of the provider, and insurance, which may be a stronger indicator of where a person seeks care than proximity alone.^6, 8^ Similarly, operating hours of care facilities and pharmacies, cost, vehicle access, public transit safety, and convenience of locations could be more of a barrier than distance to care facility itself.^2, 9^

Another factor affecting health care outcomes is accessibility to transportation, including vehicle access and mode of transportation.^9^ Moreover, transportation availability and getting transportation assistance are important factors that influence a patient’s ability to receive and seek continuous care,^10^ particularly for racial/ethnic minorities and people living in high-poverty and high HIV burden areas. Providing accessibility to HIV care resources by minimizing transportation-related barriers to care centers, improves HIV care outcomes,^1^ particularly for first time clinic visits, which lead to better engagement in sustained medical care.^10^ People living in economically-deprived areas often experience poor access to transportation, with lack of vehicle ownership and inadequate public transportation options.^10, 11^ Additionally, the effects of these structural barriers often impact racial/ethnic disparities.^1^ Racial/ethnic minorities primarily use public transportation more frequently than non-Hispanic Whites to access HIV care.^11, 12^ However, public transit routes and schedules may be restricted as they are inadequately funded^12^, requiring lengthy travel times, and lacking sufficient safety.^11^ Consequently, the lack of adequate transportation systems and reliance on public transportation for racial and ethnic minorities could negatively impact their health outcomes. An important indicator of improved health outcomes for people with HIV (PWH) is viral suppression. Achieving sustained viral suppression, defined as having < 200 copies/ml on all viral loads tests within a year,^13^ greatly reduces the transmission risk of HIV to others.^13, 14^ Recent studies that have examined sustained viral suppression demonstrate that non-Hispanic Blacks and Hispanics are less likely to achieve and maintain a consistently suppressed viral load ^13^ than non-Hispanic whites. Transportation availability and accessibility has been noted as one of the structural barrier to achieving optimal health outcomes for racial/ethnic minorities in prior studies.^9, 11, 12^ A 2020 report by Miami-Dade County on efforts to End the HIV Epidemic highlighted the county's inadequate public transportation infrastructure, identifying it as a barrier to accessing healthcare facilities and adhering to medication appointments due to the challenges of unreliable transportation for PWH.^15^ Hence, it is important to investigate the influence of distance to care facilities and transportation needs on sustained viral suppression. We hypothesize that the impact of travel distance and transportation needs on sustained viral suppression will be greater among minorities compared to non-Hispanic whites.

## Methods

### Datasets

Data were collected from the Miami-Dade County Ryan White HIV/AIDS Program (RWP) in 2017. The RWP provides medical case management, antiretroviral medication through AIDS Drug Assistance Program (ADAP) pharmacies, outpatient primary medical care, co-pay and premium support for clients with Affordable Care Act (ACA) policies (in 2017, ACA policies were only available to RWP clients with an income above 100% of the federal poverty level), and other ancillary services for low-income, uninsured PWH.^16^ To ensure that only existing clients with active RWP enrollment in 2017 and those who had received at least 1 year of RWP services were included in the analyses, we included clients receiving medical case management services (including peer education support) prior to January 1, 2017, and those who had at least one viral load service in 2017. Clients were excluded from this study if they only received ancillary services from the program (out of network clients without viral load data), had missing data on their client assessments, or whose cases had been closed due to financial ineligibility, relocation, or mortality. Five-year 2013–2017 estimates for neighborhood characteristics including poverty, employment, education, income, racial/ethnic makeup, residential movements and relocation, English language aptitude, and immigration status were collected from the American Community Survey (ACS) by ZIP Code Tabulation Areas (ZCTAs), while the homicide index was collected from Simply Analytics by ZIP Code.

### Exposure/Predictor Variables and Outcome

Street-level data for Miami-Dade County streets were collected from Miami-Dade County Open Hub^17^ and Miami-Dade County ZCTA shapefile data were collected from US Census Bureau Tiger/Line shapefiles.^18^ Due to our data user agreement, we did not have access to clients' addresses. The Miami-Dade County Ryan White Program restricted our access to only the ZIP Code of residence to protect client confidentiality. To estimate travel distance and other travel-related variables, we used the centroid of the ZIP Code Tabulation Area of the client’s residence as an approximation for clients' addresses. The locations for each RWP-funded medical case management (MCM) site and the ADAP pharmacies were geocoded using address locator in ArcGIS 10.8.1. Medical case managers (MCM) are case workers who connect clients with RWP providers to obtain HIV medications and HIV-related healthcare, including psychological and other services, aimed at improving clients' health outcomes. RWP clients visit case managers based on their needs, but the standard of care is to have some contact at least once every 90 days. Distance in miles was calculated using the closest facility function within the Network Analyst tool, utilizing the client’s residence ZCTA centroid as the starting point and their MCM site of choice as the destination. The distances to the nearest MCM site and nearest ADAP pharmacies were also calculated from each resident’s ZCTA centroid. Travel-related variables were dichotomized into: (yes/no travel to the nearest MCM facility), (yes/no traveled greater than or less than the median travel distance of 7.2 miles to MCM site of choice, and yes/no traveled greater than or less than the median travel distance of 8.0 miles to nearest ADAP pharmacy). Finally, a 5-mile distance buffer radius,^6^ for both the MCM sites and ADAP pharmacies were calculated using the service area function of the Network Analyst tool.

Demographic data abstracted from the RWP included race/ethnicity, age, gender, US nativity, mode of HIV exposure, history of AIDS diagnosis, recipient of RWP subsidized ACA health care, household income in federal poverty level (FPL), total number of clients that physician has, and having access to transportation for health care/dental/social service appointments (including both personal and public transportation). Race/ethnicity was categorized as Non-Hispanic Black (NHB) (excluding Haitians), Non-Hispanic White, Hispanic, and Haitian. Clients were asked about their race, ethnicity, and preferred language to determine these categories. Given the large Haitian population with HIV in Florida, particularly in Miami-Dade County, clients were specifically asked if they were Haitian. The preferred language variable helped avoid overlapping ethnic categories. Clients were first grouped as Haitian, Hispanic, or Other based on their responses and preferred language. Then, the race variable was used to classify them as Black, White, or Other. Finally, ethnicity and race were combined to categorize clients as Non-Hispanic Black (excluding Haitians), Haitian, Non-Hispanic White, or Hispanic. Furthermore, three psychosocial indices (mental health index, substance use index, and income/SES index) from RWP dataset and three neighborhood indices (socioeconomic [SES] disadvantage index, residential instability index, racial/language homogeneity index) from ACS.^19^ Variables for the psychosocial indices were chosen based on the Andersen Behavioral Model for HIV Health Service Utilization,^20, 21^ while neighborhood indices variables were selected based on Social Disorganization theory.^22^ Details on the creation of the indices are explained elsewhere,^19^ but briefly, exploratory and confirmatory factor analyses were used to develop psychosocial and neighborhood indices. For psychosocial indices, 16 variables were selected, while for neighborhood indices, 24 variables were selected. There was no collinearity observed among the variables in the model. All indices showed strong internal consistency and were categorized into tertiles. A linear combination of variables’ observed weights was calculated for each index.

The outcome variable was sustained viral suppression, defined as having < 200 copies/ml for all viral load test results in 2017. If a client had any of their viral loads ≥ 200 copies/ml in 2017, they were not considered suppressed. For clients who had only one suppressed viral load test in 2017, or all suppressed tests were within a three-month period, we also reviewed their last viral load test from 2016 to determine if their viral suppression was sustained.^23^

## Statistical Analysis

All statistical analyses were performed using SAS 9.4. Summary statistics were computed using the Chi-square test for categorical variables and Wilcoxon rank-sum test for continuous variables. This was followed by four separate multilevel logistic regression models. We first ran an unconditional model to measure the intraclass correlation (ICC) using a random intercept model with ZIP code random effects. Next, we only included individual-level variables (demographic, travel distance variables, having access to transportation, and psychosocial indices). The third model included the individual-level variables from the second model and neighborhood-level variables. The final model included the variables from the third model and interaction terms between the travel distance variables and race/ethnicity as well as having access to transportation and race/ethnicity. The models were analyzed separately for MCM site and ADAP pharmacies. Additionally, maps of Miami-Dade County illustrating a 5-mile service buffer radius were generated for MCM sites and ADAP pharmacies using ArcMap 10.8.1.

### Research Ethics Approval

This study was approved by the Florida International University Institutional Review Board (#190406).

## Results

A total of 6180 clients in the RWP were included in this analysis. Race/ethnicity, US nativity, total number of clients that physician has, having access to transportation, and SES disadvantage index were significantly associated with travel to nearest MCM facility (Table 2). Most (88.9%) clients did not seek care at a MCM site that was closest to their residence. Table 2 also shows the median travel distance to client’s MCM site of choice and median distance to client’s nearest ADAP pharmacy. The median travel distance for all clients to the MCM sites of their choice and nearest ADAP pharmacy was 7.2 miles and 8.0 miles, respectively. Clients who were Haitian, foreign born, enrolled in an ACA health plan and had access to transportation traveled the longest over these thresholds. Distribution of the service providers and the five mile-distance buffer radii from the MCM site and ADAP pharmacies are displayed in Fig. 1.

Results for the multilevel logistic regression models are shown in Table 3 for MCM site. The unconditional random intercept model determined an ICC of 0.04, indicating that 4% of the variation in achieving sustained viral suppression is due to characteristics at the neighborhood level. While the value of the ICC was small, studies have shown that even an ICC of 5% shows important geographic variations.^24^ Younger individuals, NHB and Haitians, those with household incomes < 100% of the FPL, and those previously diagnosed with AIDS had a lower odds of achieving sustained viral suppression. Moreover, clients who did not know their provider’s name and those with higher psychosocial indices value (poor mental health, high substance use, and low income/SES) also had a lower odd of achieving sustained viral suppression (Supplemental Table 1). On the other hand, clients enrolled in an ACA health plan had higher odds of sustained viral suppression than those not in ACA.

None of the neighborhood indices were significantly associated with sustained viral suppression in Model 3 (Supplemental Table 1). In Model 4, the overall moderating effect of race/ethnicity on the association between travel-variables and sustained viral suppression was not significant. However, we observed significant results for race/ethnicity conditional on travel-related variables and access to transportation. Haitians had lower odds of achieving sustained viral suppression compared to NHW among those who have access to transportation (adjusted odds ratio [aOR]: 0.53; 95% confidence interval [CI]:0.35–0.80). Among clients who do not have access to transportation NHB (0.28; 0.10–0.76), Hispanics (0.35; 0.13–0.94), and Haitians (0.16; 0.05–0.52) had lower odds of achieving sustained viral suppression when compared to NHW. Non-Hispanic Blacks (0.66; 0.46–0.94) and Haitians (0.46: 0.31–0.70) had lower odds of sustained viral suppression compared to NHW among clients that did not travel to their nearest MCM site. We did not observe statistically significant racial/ethnic disparities among clients who traveled to their nearest MCM. Among clients who traveled more than the median distance of 7.2 miles to their MCM, NHB and Haitians also exhibited lower odds of sustained viral suppression (0.55; 0.36–0.84 and 0.39; 0.24–0.66, respectively) when compared to NHW. No racial/ethnic disparities were observed among clients that traveled less than or equal to the median distance of 7.2 miles to their MCM site.

Table 4 shows the results of the multilevel model for ADAP pharmacies analysis. The ADAP pharmacies models 1–3 results (Supplemental Table 2) were similar to those of the MCM site models. In Model 4, we observed that among those that traveled more than 8.0 miles (median) to their nearest ADAP pharmacy, NHB compared to NHWs had lower odds of sustained viral suppression (0.48: 0.29–0.79). Lastly, Haitians (0.50; 0.30–0.80) had lower odds of sustained viral suppression when compared to NHW among clients who traveled less than or equal to the median distance of 8.0 miles to their nearest ADAP pharmacy (Table 3).

## Discussion

This study sought to investigate the association between travel distance and transportation needs and sustained viral suppression and the moderating effect of race/ethnicity in this association. Our analysis suggests four primary findings. First, our study noted that most clients (88.9%) did not use the MCM facility that was closest to their home. Second, racial/ethnic minorities had lower odds of sustained viral suppression when compared to NHW among clients who did not have access to transportation. Third, NHB’s and Haitians had lower odds of sustained viral suppression compared to NHW if they did not use their nearest MCM site and if they traveled farther than the median distance of 7.2 miles to seek care. Finally, among clients that traveled less than or equal to the median distance of 8.0 miles to their nearest ADAP pharmacy, Haitians had lower odds of sustained viral suppression compared to NHW, and among clients that traveled more than the median 8.0 miles to their nearest ADAP pharmacy to receive their medication, NHB had lower odds of sustained viral suppression than NHW.

In our study, RWP clients traveled farther than their nearest care facility to receive care. Previous studies have also found that PWH seek care from facilities that are farther than the closest one from their residence.^6, 8^ These studies suggest that PWH might travel far due to needing facilities that provide a variety of ancillary services, seeking care at multiple facilities for other comorbid conditions, provider preferences, and fear of stigma or disclosure of HIV status.^4, 6, 8^ Additionally, at the time when the study data were generated, here were only two ADAP pharmacies located in Miami-Dade County, and access to these pharmacies was influenced by hours of operation, delivery service options, and convenience of location.^2^ Further studies, particularly qualitative studies, are needed to determine factors that are influencing PWH to seek care away from their nearest facility.

Among clients that do not have access to transportation, all racial/ethnic minorities had lower odds of sustained viral suppression when compared to NHWs. Conversely, Hispanics and NHB that had access to transportation did not differ from NHW in sustained viral suppression. Racial/ethnic minorities face various challenges including residing in low socioeconomic neighborhoods,^25^ which can impact their health. A study has shown that transportation accessibility including vehicle ownership and access to permanent and consistent means of transportation, particularly in low socioeconomic neighborhoods was significantly associated with retention in care and viral suppression, respectively.^10^ However having inadequate access to transportation combined with longer distance to care facilities is a barrier to care.^26^ Hence, lacking access to transportation might be influencing racial/ethnic minorities from achieving a consistently suppressed viral load.

Particularly Haitians and NHBs had lower odds of achieving sustained viral suppression if they traveled farther away from nearest MCM site and traveled beyond the median travel distances of 7.2 miles to clients’ MCM site of choice, when compared to NHWs. We did not observe these differences for NHB and Haitian clients that traveled to their nearest MCM site or for those that traveled less than the median travel distance threshold. In addition to transportation needs, Haitian immigrants and Haitian Americans face numerous challenges, including stigma and fear of disclosure to family, friends, and society; low socioeconomic status; legal and sociopolitical climate; cultural barriers and belief systems; low education and literacy levels; and language barriers, which serve as barriers to achieving optimal HIV care outcomes.^27, 28^ Thus, there might be unmeasured confounding from factors such as stigma and fear of disclosure, which we could not measure in our data. Among Caribbean born immigrants in Florida, Haitians were less likely to be retained in care and achieve viral suppression.,^29^ Additionally, NHBs have a higher prevalence of HIV than NHW, and are less likely to achieve viral suppression and sustained viral suppression.^13, 30^ Hence, traveling longer distance to care facilities might be a contributing factor to the poor health outcomes of NHBs and Haitians.

Differences in sustained viral suppression were observed for NHB’s and Haitians accessing ADAP pharmacies. We observed that NHBs exhibited lower odds of sustained viral suppression when traveling longer than the median travel distance of 8.0 miles to their nearest ADAP pharmacy, while Haitians had lower odds of sustained viral suppression if they traveled 8.0 miles or less. Factors influencing poor sustained viral suppression for NHB’s traveling longer distance might include transportation cost^31^ and accessibility to pharmacies (hours of operation, convenience and safety of pharmacy location),^2^ which affects medication adherence. A qualitative study conducted among clients from RWP clinics in Philadelphia found that fear of HIV status disclosure in care facilities including pharmacies was a barrier for PWH.^32^ With only two ADAP pharmacies providing HIV medication, going to an “out-of-neighborhood” pharmacy could involve considerable travel inconvenience. In the years since the data were collected (and especially since the COVID Pandemic), ADAP pharmacy home delivery service has improved, and ADAP has diversified its retail outlets to include several CVS Specialty Pharmacies that provide local retail services and home delivery options. Some studies have shown home delivery service rather than traditional pharmacy delivery systems serve as facilitators to medication adherence.^2, 11^

This study has several limitations. First, our analysis utilized the centroid of the client’s ZCTA of residence to measure distance to care facilities, which limits our precision for our distance variables as not all clients live in or near the centroid of a ZCTA. Clients in the same ZCTA that go to the same location are assigned the same travel distance regardless of race/ethnicity. Moreover, using ZIP codes to define neighborhoods can result in spatial misclassification, as individuals interact with multiple neighborhoods beyond the ZIP code boundaries. Hence, the analysis reduced the variation of travel distance compared to the actual distance. Second, we only included travel from a residential ZCTA to care facilities and did not consider that client’s origin of transportation might be from other locations including workplaces. Third, since we did not have data to identify which of the two ADAP pharmacies were being used by RWP clients. This might not accurately reflect the distance which clients traveled to access their medication in 2017.Lastly, our distance calculation did not factor in the use of public transportation routes or actual mode of transportation used by clients, which might increase travel distance and time to MCM sites and ADAP pharmacies.

## Conclusion

Having reliable and consistent transportation to receive RWP services and to obtain HIV medication is an important facilitator to achieve optimal health outcomes. This study identified that clients who did not have access to transportation had poor sustained viral suppression rates, and these differences were magnified for racial/ethnic minorities. Additionally, longer travel distance disproportionally affected minorities, particularly Haitians and NHB’s. Addressing transportation barriers to care by examining the feasibility of telehealth might alleviate the challenges faced by racial/ethnic minorities and minimize disparities. Furthermore, clients, in general, chose a facility that was not closest to their place of residence, indicating that other factors besides travel distance might be important indicators of where clients access care. Other unmeasured individual and structural factors such as structural stigma and provider preference may have a greater influence on racial/ethnic minority difference in sustained viral suppression. Hence, future studies should examine these factors to work towards ending the HIV epidemic.

## Figures and Tables

**Table 1:** Variables and their respective level in the multilevel mixed effects modes

Level	Variable
Level 1 (Outcomes)	Sustained viral suppression
Level 1 (Moderator)	Travel related variables: travel distance to the nearest MCM facility of care traveled greater than or less than the median travel distance of 7.2 miles to MCM site of choice traveled greater than or less than the median travel distance of 8.0 miles to nearest ADAP pharmacy access to transportation
Level 1 (Predictor)	race/ethnicity
Level 1 (covariates)	age gender, US nativity mode of HIV exposure history of AIDS diagnosis recipient of RWP subsidized ACA health care household income in federal poverty level (FPL) total number of clients that physician has three psychosocial indices (mental health index, substance use index, and income/SES index)
Level 2 (Covariates)	three neighborhood indices (socioeconomic [SES] disadvantage index, residential instability index, racial/language homogeneity index)

**Table 2:** Characteristics of RWP clients by travel distance variables in Miami Dade County, Florida

Characteristics	Travel to nearest MCM		P-value	Median travel distance to MCM of choice in miles (Median, IQR)	Median travel distance to nearest ADAP in miles (Median, IQR)
	Yes	No			
**Total**	687	5493		7.2 (0.35–35.6)	8.0 (0.54–31.6)
**Race/Ethnicity**			**< 0.0001**		
NHB	205 (29.8)	1281 (23.3)		5.47 (3.29–9.40)	7.48 (3.85–11.28)
Hispanic	372 (54.1)	3241 (59.0)		8.38 (4.08–13.2)	7.48 (3.26–10.97)
Haitian	44 (6.5)	638 (11.6)		8.56 (5.47–11.49)	11.28 (8.26–13.29)
NHW	66 (9.6)	333 (6.1)		5.82 (3.29–9.74)	8.02 (6.85–10.97)
**Age**			0.1247		
18–34	163 (23.7)	1172 (21.3)		7.35 (3.83–12.04)	8.02 (4.39–11.28)
35–49	275 (40.0)	2121 (38.6)		7.21 (3.67–11.77)	8.02 (4.39–11.28)
≥50	249 (36.3)	2200 (40.1)		7.07 (3.67–11.83)	8.02 (4.66–11.28)
**Gender**			0.5708		
Males	521 (75.8)	4219 (76.8)		7.21 (3.68–12.04)	7.48 (4.39–11.16)
Female	166 (24.2)	1274 (23.2)		6.64 (3.67–11.36)	8.26 (6.05–11.28)
**US Born**			**< 0.0001**		
No	400 (58.2)	3824 (69.6)		8.38 (4.53–12.51)	8.02 (4.39–11.28)
Yes	287 (41.8)	1669 (30.4)		5.61 (3.38–9.76)	7.48 (5.38–10.99)
**Household Income in FPL**			0.2200		
> 200%	177 (25.7)	1263 (23.0)		7.66 (4.12–12.24)	8.14 (4.53–11.28)
100–199%	244 (35.5)	1960 (35.7)		7.21 (3.95–12.17)	8.02 (5.38–11.28)
<100%	266 (38.7)	2270 (41.3)		6.78 (3.61–11.35)	7.48 (4.39–11.28)
**Mode of HIV Transmission**			0.1928		
Heterosexual	287 (41.8)	2476 (45.0)		6.78 (3.64–11.34)	8.26 (5.66–11.28)
MSM	370 (53.9)	2821 (51.4)		7.44 (3.83–12.41)	7.48 (4.39–10.97)
IDU & Other	30 (4.3)	196 (3.6)		7.39 (4.15–12.07)	8.02 (4.39–11.28)
**History of AIDS Diagnosis**			0.5883		
No	411 (59.8)	3227 (58.8)		7.21 (3.86–12.17)	8.02 (4.66–11.28)
Yes	276 (40.2)	2266 (41.2)		7.03 (3.63–11.68)	8.02 (4.66–11.28)
**Total number of Clients that Physician has**			**0.0044**		
1–99	152 (22.1)	1459 (26.5)		7.23 (4.60-12.17)	8.69 (6.05–11.28)
100–199	222 (32.3)	1614 (29.4)		6.47 (3.61–11.28)	8.02 (5.66–11.28)
≥200	261 (38.0)	2137 (38.9)		7.56 (3.67–12.20)	7.48 (3.26–10.99)
Unknown Physician name	52 (7.6)	283 (5.2)		6.60 (3.48–11.35)	8.02 (4.39–11.28)
**Enrolled in ACA Health Care Plan**			0.7023		
No	579 (84.2)	4660 (84.8)		7.07 (3.67–11.77)	8.02 (4.66–11.28)
Yes	108 (15.7)	833 (15.2)		7.68 (4.69–12.47)	8.26 (4.39–11.28)
Access to Transportation			**< 0.0001**		
Yes	593 (86.3)	5044 (91.8)		7.21 (3.71–11.93)	8.02 (4.66–11.28)
No	94 (13.7)	449 (8.2)		6.94 (3.43–11.22)	7.48 (3.46–10.97)
**Sustained Viral Suppression**			0.3239		
No	119 (17.3)	1037 (18.9)		6.26 (3.58–10.59)	8.02 (4.66–11.28)
Yes	568 (82.7)	4456 (81.1)		7.33 (3.83–12.11)	8.02 (4.66–11.28)
**Psychosocial Indices** ^a^					
**Mental health index**					
Mean and SD	0.05; (1.04)	−0.01; (0.99)			
Median and IQR	−0.49; (−0.48:0.88)	−0.48; (−0.48: −0.48)	0.0692		
**Substance use index**					
Mean and SD	0.02; (1.06)	−0.003; (0.99)			
Median and IQR	−0.25; (−0.25: −0.25)	−0.25); (−0.25: −0.25)	0.9795		
**Income/SES index**					
Mean and SD	−0.03; (0.99)	0.003; (1.00)			
Median and IQR	−0.67; (−0.67: 0.70)	−0.67; (−0.67: 0.70)	0.3185		
**Neighborhood indices** ^a^					
**SES disadvantage index**					
Mean and SD	0.30; (0.80)	0.57; (0.86)			
Median and IQR	0.29; (−0.35: 0.79)	0.76; (−0.13: 1.37)	**< 0.0001**		
**Residential instability index**					
Mean and SD	0.45; (0.87)	0.43; (0.90)			
Median and IQR	0.51; (−0.32: 1.36)	0.33; (−0.29: 1.09)	0.9584		
**Racial/language homogeneity index**					
Mean and SD	0.26; (0.95)	0.24; (1.15)			
Median and IQR	0.28; (−0.28: 0.98)	0.28; (−0.52: 1.18)	0.8347		
**Homicide index**					
Mean and SD	104.37; (45.05)	105.43 (44.88)			
Median and IQR	101.8; (65.6: 151.6)	101.8; (68.0: 148.2)	0.2847		

NHB: Non-Hispanic Black; NHW: Non-Hispanic White; MSM: Men who have sex with men; FPL: Federal Poverty Level; IDU: Injection drug use; ACA: Affordable Care Act; SES: socioeconomic; MCM: Medical case management; ADAP: AIDS Drug Assistance Program

a = A higher index score for each index indicates increased social disadvantage/worse conditions

**Table 3:** Multilevel mixed effects models for sustained viral suppression for Ryan White HIV/AIDS Program Clients by Race/ethnicity and Travel distance variables to Medical Case Management Sites (MCM) in Miami-Dade County, Florida, 2017

	Model 1: Crude/unadjusted			Model 4: Access to transportation* race/ethnicity			Model 4: Nearest MCM*race/ethnicity			Model 4: Median distance to MCM*race/ethnicity		
	OR	95% CI		OR	95% CI		OR	95% CI		OR	95% CI	
**Race/Ethnicity**												
NHB vs. NHW	**0.50**	**0.37**	**0.68**	**0.44**	**0.26**	**0.76**	**0.55**	**0.34**	**0.90**	**0.64**	**0.44**	**0.92**
Hispanic vs. NHW	1.15	0.86	1.54	0.59	0.34	1.01	0.75	0.46	1.23	0.91	0.63	1.30
Haitian vs. NHW	**0.53**	**0.38**	**0.75**	**0.29**	**0.16**	**0.55**	**0.59**	**0.31**	**1.12**	**0.48**	**0.32**	**0.73**
**Age**												
18–34 vs. 50+	**0.50**	**0.42**	**0.59**	**0.38**	**0.31**	**0.46**	**0.37**	**0.31**	**0.45**	**0.38**	**0.31**	**0.46**
35–49 vs. 50+	**0.69**	**0.59**	**0.81**	**0.55**	**0.47**	**0.65**	**0.55**	**0.47**	**0.65**	**0.55**	**0.47**	**0.65**
**Gender**												
Women vs. Men	**0.84**	**0.72**	**0.97**	1.11	0.92	1.34	1.12	0.93	1.35	1.11	0.92	1.34
**US Born**																																																																																																																																		
Yes vs. No	**0.57**	**0.49**	**0.65**	0.85	0.68	1.05	0.84	0.68	1.05	0.85	0.68	1.05
**Household Income in FPL**												
100–199% vs. ≥200%	**0.68**	**0.55**	**0.83**	**0.77**	**0.62**	**0.95**	**0.77**	**0.62**	**0.95**	**0.76**	**0.62**	**0.95**
< 100% vs. ≥200%	**0.35**	**0.29**	**0.42**	**0.54**	**0.44**	**0.67**	**0.54**	**0.44**	**0.67**	**0.54**	**0.44**	**0.67**
**Mode of HIV Transmission**												
Heterosexual vs.MSM	**0.71**	**0.61**	**0.82**	0.87	0.72	1.06	0.87	0.72	1.06	0.87	0.72	1.06
IDU vs. MSM	**0.51**	**0.37**	**0.70**	0.76	0.54	1.08	0.77	0.54	1.09	0.77	0.54	1.09
**History of AIDS Diagnosis**												
Yes vs. No	**0.59**	**0.52**	**0.67**	**0.55**	**0.48**	**0.63**	**0.55**	**0.47**	**0.63**	**0.55**	**0.48**	**0.63**
**Total number of Clients that Physician Has**												
1–99 vs. ≥200	**0.74**	**0.63**	**0.88**	0.88	0.73	1.05	0.88	0.73	1.05	0.87	0.73	1.05
100–199 vs ≥ 200	**0.77**	**0.66**	**0.91**	0.93	0.78	1.11	0.93	0.78	1.11	0.93	0.78	1.11
Unknown Physician name vs. ≥200	**0.48**	**0.36**	**0.62**	**0.63**	**0.47**	**0.84**	**0.63**	**0.47**	**0.85**	**0.63**	**0.47**	**0.85**
**Enrolled in ACA Health Care Plan**												
Yes vs. No	**2.21**	**1.76**	**2.77**	**1.37**	**1.08**	**1.74**	**1.37**	**1.08**	**1.75**	**1.37**	**1.08**	**1.75**
**Access to Transportation**												
No vs. Yes	**0.64**	**0.52**	**0.79**	0.85	0.61	1.17	**0.70**	**0.56**	**0.88**	**0.72**	**0.57**	**0.90**
**Travel to Nearest MCM**												
No vs. Yes	0.97	0.78	1.20	0.82	0.65	1.03	**0.66**	**0.47**	**0.92**	0.83	0.66	1.04
**Travel Distance to MCM (median distance = 7.2 miles)**												
>7.2 miles vs. ≤7.2 miles	**1.24**	**1.06**	**1.44**	1.16	0.97	1.38	1.16	0.97	1.39	1.10	0.88	1.36
**Psychosocial Indices** ^a^												
Mental health index	**0.76**	**0.72**	**0.81**	**0.85**	**0.80**	**0.91**	**0.85**	**0.80**	**0.91**	**0.85**	**0.80**	**0.91**
Substance use index	**0.78**	**0.74**	**0.83**	**0.89**	**0.84**	**0.95**	**0.89**	**0.84**	**0.95**	**0.89**	**0.84**	**0.95**
Income/SES index	**0.69**	**0.66**	**0.74**	**0.84**	**0.78**	**0.90**	**0.84**	**0.78**	**0.90**	**0.84**	**0.78**	**0.90**
**Neighborhood Indices** ^a^												
SES disadvantage index	**0.80**	**0.71**	**0.91**	0.98	0.88	1.09	0.98	0.87	1.09	0.98	0.88	1.09
Residential instability index	**0.84**	**0.74**	**0.96**	0.94	0.82	1.07	0.93	0.81	1.07	0.93	0.81	1.07
Racial/language homogeneity index	**0.77**	**0.71**	**0.84**	0.94	0.86	1.02	0.94	0.86	1.02	0.94	0.86	1.02
Homicide index	1.00	1.00	1.00	1.00	1.00	1.00	1.00	1.00	1.00	1.00	1.00	1.00
**Interactions for subgroups**												
Access to Transport (Yes)												
(NHB vs. NHW)				0.70	0.50	1.00						
(Hispanics vs. NHW)				1.01	0.71	1.43						
**(Haitian vs. NHW)**				**0.53**	**0.35**	**0.80**						
Access to Transport (No)												
**(NHB vs. NHW)**				**0.28**	**0.10**	**0.76**						
**(Hispanics vs. NHW)**				**0.35**	**0.13**	**0.94**						
**(Haitian vs. NHW)**				**0.16**	**0.05**	**0.52**						
Travel to Nearest MCM (Yes)												
(NHB vs. NHW)							0.46	0.19	1.12			
(Hispanics vs. NHW)							0.60	0.25	1.47			
(Haitian vs. NHW)							0.75	0.23	2.47			
Travel to Nearest MCM (No)												
**(NHB vs. NHW)**							**0.66**	**0.46**	**0.94**			
(Hispanics vs. NHW)							0.94	0.66	1.34			
**(Haitian vs. NHW)**							**0.46**	**0.31**	**0.70**			
Distance to MCM (median distance > 7.2 Miles)												
**(NHB vs. NHW)**										**0.55**	**0.36**	**0.84**
(Hispanics. NHW)										0.79	0.52	1.22
**(Haitian vs. NHW)**										**0.39**	**0.24**	**0.66**
Distance to MCM (median distance ≤ 7.2 Miles)												
(NHB vs. NHW)										0.77	0.46	1.27
(Hispanics vs. NHW)										1.06	0.65	1.73
(Haitian vs. NHW)										0.58	0.34	1.00

NHB: Non-Hispanic Black; NHW: Non-Hispanic White; MSM: Men who have sex with men; FPL: Federal Poverty Level; IDU: Injection drug use; ACA: Affordable Care Act; SES: socioeconomic; MCM: Medical case management; ADAP: AIDS Drug Assistance Program

a = A higher index score for each index indicates increased social disadvantage/worse conditions

**Table 4:** Multilevel mixed effects models for sustained viral suppression for Ryan White HIV/AIDS Program Clients by Race/ethnicity and travel distance variables to AIDS Drug Assistance Program (ADAP) in Miami-Dade County, Florida, 2017

	Model 4: Access to transportation* race/ethnicity			Model 4: ADAP* race/ethnicity		
	OR	95% CI		OR	95% CI	
**Race/Ethnicity**						
NHB vs. NHW	**0.44**	**0.26**	**0.76**	**0.64**	**0.46**	**0.90**
Hispanic vs. NHW	0.60	0.35	1.02	0.90	0.64	1.26
Haitian vs. NHW	**0.30**	**0.16**	**0.56**	**0.51**	**0.34**	**0.77**
**Age**						
18–34 vs. 50+	**0.38**	**0.31**	**0.46**	**0.37**	**0.31**	**0.46**
35–49 vs. 50+	**0.56**	**0.47**	**0.65**	**0.55**	**0.47**	**0.65**
**Gender**						
Women vs. Men	1.12	0.93	1.34	1.12	0.93	1.35
**US Born**						
Yes vs. No	0.85	0.68	1.05	0.86	0.69	1.06
**Household Income in FPL**						
100–199 vs. ≥200%	**0.76**	**0.62**	**0.95**	**0.76**	**0.62**	**0.94**
<100 vs. ≥200%	**0.54**	**0.44**	**0.67**	**0.54**	**0.43**	**0.67**
**Mode of HIV Transmission**						
Heterosexual vs. MSM	0.87	0.71	1.06	0.87	0.72	1.06
IDU vs. MSM	0.76	0.54	1.08	0.76	0.54	1.08
**History of AIDS Diagnosis**						
Yes vs. No	**0.55**	**0.48**	**0.64**	**0.55**	**0.48**	**0.64**
**Total number of Clients that Physician has**						
1–99 vs. ≥200	0.88	0.73	1.05	0.88	0.73	1.05
100–199 vs ≥ 200	0.93	0.78	1.10	0.93	0.78	1.11
Unknown Physician name vs. ≥200	**0.63**	**0.47**	**0.85**	**0.63**	**0.47**	**0.84**
**Enrolled in ACA Health Care Plan**						
Yes vs. No	**1.36**	**1.07**	**1.73**	**1.37**	**1.08**	**1.74**
**Access to Transportation**						
No vs. Yes	0.86	0.62	1.19	**0.73**	**0.58**	**0.91**
**Travel Distance to nearest ADAP (median distance = 8.0 miles)**						
>8.0 miles vs. ≤8.0miles	0.98	0.81	1.18	0.84	0.66	1.06
**Psychosocial Indices** ^a^						
Mental Health index	**0.85**	**0.80**	**0.91**	**0.85**	**0.80**	**0.91**
Substance Use index	**0.89**	**0.84**	**0.94**	**0.89**	**0.84**	**0.95**
Income/SES index	**0.84**	**0.78**	**0.90**	**0.84**	**0.78**	**0.90**
**Neighborhood Indices** ^a^						
SES Disadvantage index	0.97	0.87	1.09	0.97	0.87	1.09
Residential Instability index	0.94	0.82	1.07	0.94	0.82	1.08
Racial/Language Homogeneity index	0.93	0.86	1.01	0.93	0.85	1.01
Homicide index	1.00	1.00	1.00	1.00	1.00	1.00
**Interactions for subgroups**						
Access to Transport (Yes)						
(NHB vs. NHW)	0.70	0.50	1.00			
(Hispanics vs. NHW)	1.00	0.71	1.42			
**(Haitian vs. NHW)**	**0.53**	**0.35**	**0.79**			
Access to Transport (No)						
**(NHB vs. NHW)**	**0.28**	**0.10**	**0.77**			
**(Hispanics vs. NHW)**	**0.36**	**0.13**	**0.95**			
**(Haitian vs. NHW)**	**0.17**	**0.05**	**0.54**			
Distance to ADAP (median distance > 8.0 Miles)						
**(NHB vs. NHW)**				**0.48**	**0.29**	**0.79**
(Hispanics vs. NHW)				0.73	0.45	1.20
(Haitian vs. NHW)				0.56	0.29	1.07
Distance to ADAP (median distance ≤ 8.0 Miles)						
(NHB vs. NHW)				0.79	0.50	1.26
(Hispanics vs. NHW)				1.04	0.66	1.64
**(Haitian vs. NHW)**				**0.50**	**0.31**	**0.80**

NHB: Non-Hispanic Black; NHW: Non-Hispanic White; MSM: Men who have sex with men; FPL: Federal Poverty Level; IDU: Injection drug use; ACA: Affordable Care Act; SES: socioeconomic; MCM: Medical case management; ADAP: AIDS Drug Assistance Program

a = A higher index score for each index indicates increased social disadvantage/worse conditions
